# Psychological burden predicts new-onset diabetes in men: A longitudinal observational study in the Fukushima Health Management Survey after the Great East Japan earthquake

**DOI:** 10.3389/fendo.2022.1008109

**Published:** 2022-12-02

**Authors:** Hiroyuki Hirai, Masanori Nagao, Tetsuya Ohira, Masaharu Maeda, Kanako Okazaki, Hironori Nakano, Fumikazu Hayashi, Mayumi Harigane, Yuriko Suzuki, Atsushi Takahashi, Akira Sakai, Junichiro J. Kazama, Mitsuaki Hosoya, Hirooki Yabe, Seiji Yasumura, Hitoshi Ohto, Kenji Kamiya, Michio Shimabukuro

**Affiliations:** ^1^ Department of Diabetes, Endocrinology and Metabolism, Fukushima Medical University School of Medicine, Fukushima, Japan; ^2^ Department of Internal Medicine, Shirakawa Kosei General Hospital, Fukushima, Japan; ^3^ Radiation Medical Science Center for the Fukushima Health Management Survey, Fukushima, Japan; ^4^ Department of Epidemiology, Fukushima Medical University School of Medicine, Fukushima, Japan; ^5^ Department of Disaster Psychiatry, Fukushima Medical University School of Medicine, Fukushima, Japan; ^6^ Department of Physical Therapy, Fukushima Medical University School of Health Sciences, Fukushima, Japan; ^7^ Department of Public Health, Fukushima Medical University School of Medicine, Fukushima, Japan; ^8^ Department of Adult Mental Health, National Institute of Mental Health, National Center of Neurology and Psychiatry, Tokyo, Japan; ^9^ Department of Gastroenterology, Fukushima Medical University School of Medicine, Fukushima, Japan; ^10^ Department of Nephrology and Hypertension, Fukushima Medical University School of Medicine, Fukushima, Japan; ^11^ Department of Pediatrics, Fukushima Medical University School of Medicine, Fukushima, Japan; ^12^ Department of Neuropsychiatry, Fukushima Medical University School of Medicine, Fukushima, Japan

**Keywords:** disaster, psychological stress, post-traumatic stress disorder, depression, gender differences, type 2 diabetes mellitus 4

## Abstract

**Background:**

The burden of psychological distress and post-traumatic stress disorder (PTSD) has been suggested as a factor in developing type 2 diabetes mellitus. However, longitudinal features in psychological distress- and PTSD-related new-onset diabetes mellitus have not been thoroughly evaluated.

**Methods:**

The association between probable depression and probable PTSD and the risk of developing new-onset diabetes mellitus was evaluated in a 7-year prospective cohort of evacuees of the Great East Japan Earthquake in 2011. Probable depression was defined as a Kessler 6 scale (K6) ≥ 13 and probable PTSD as a PTSD Checklist—Stressor-Specific Version (PCL-S) ≥ 44.

**Results:**

The log-rank test for the Kaplan–Meier curve for new-onset diabetes mellitus was significant between K6 ≥ 13 vs. < 13 and PCL-S ≥ 44 vs. < 44 in men but not in women. In men, both K6 ≥ 13 and PCL-S ≥ 44 remained significant in the Cox proportional hazards model after multivariate adjustment for established risk factors and disaster-related factors, including evacuation, change in work situation, sleep dissatisfaction, and education.

**Conclusion:**

The post-disaster psychological burden of probable depression and probable PTSD was related to new-onset diabetes in men but not in women. In post-disaster circumstances, prevention strategies for new-onset diabetes might consider sex differences in terms of psychological burden.

## Introduction

Psychological distress has been reported to be a risk factor for developing type 2 diabetes mellitus (1–3). States of psychological distress, such as non-specific symptoms of stress, anxiety, depression, personal traits, and type of external or psychological stressors, might play various roles in the development of diabetes mellitus (3). There is evidence that depression is an independent risk factor for type 2 diabetes mellitus (4); however, the risk of non-specific symptoms of stress are equivocal (5–7). It is also less clear whether post-traumatic stress disorder (PTSD) is associated with a higher risk of developing type 2 diabetes mellitus (8).

Previous reports of sex differences in the development of diabetes associated with psychological distress yielded conflicting results; Eriksson et al. (9) observed associations in men and others (10–12) in women. To our knowledge, there are no robust longitudinal studies comparing sex differences in the effects of PTSD on new-onset diabetes mellitus (13–16). O’Donnell claimed that the predominance of PTSD studies in male veterans generates concerns about generalizability to non-military populations or other populations defined by sex (17).

The Great East Japan Earthquake and subsequent tsunami and Fukushima Daiichi nuclear disaster, which occurred in March 2011, caused a devastating catastrophe in East Japan, mostly affecting local residents. The Fukushima Health Management Survey was conducted to investigate the effects of long-term, low-dose radiation exposure caused by the accident to assess the physical and mental well-being of evacuees (18, 19). Among the potential health concerns that arose after the Great East Japan Earthquake were mental health problems, including post-traumatic stress response, chronic anxiety and guilt, ambiguous loss, family and community separation, and stigmatization (20). A recent meta-analysis reported a higher rate of new-onset diabetes mellitus among disaster survivors (21). However, the longitudinal effects of the psychological burden on the onset of diabetes have not been elucidated in this population.

We evaluated the association between probable depression and probable PTSD and the risk of developing new-onset diabetes mellitus and its sex differences in a 7-year prospective cohort of survivors of the Great East Japan Earthquake.

## Methods

### Study design and population

**Figure 1:**
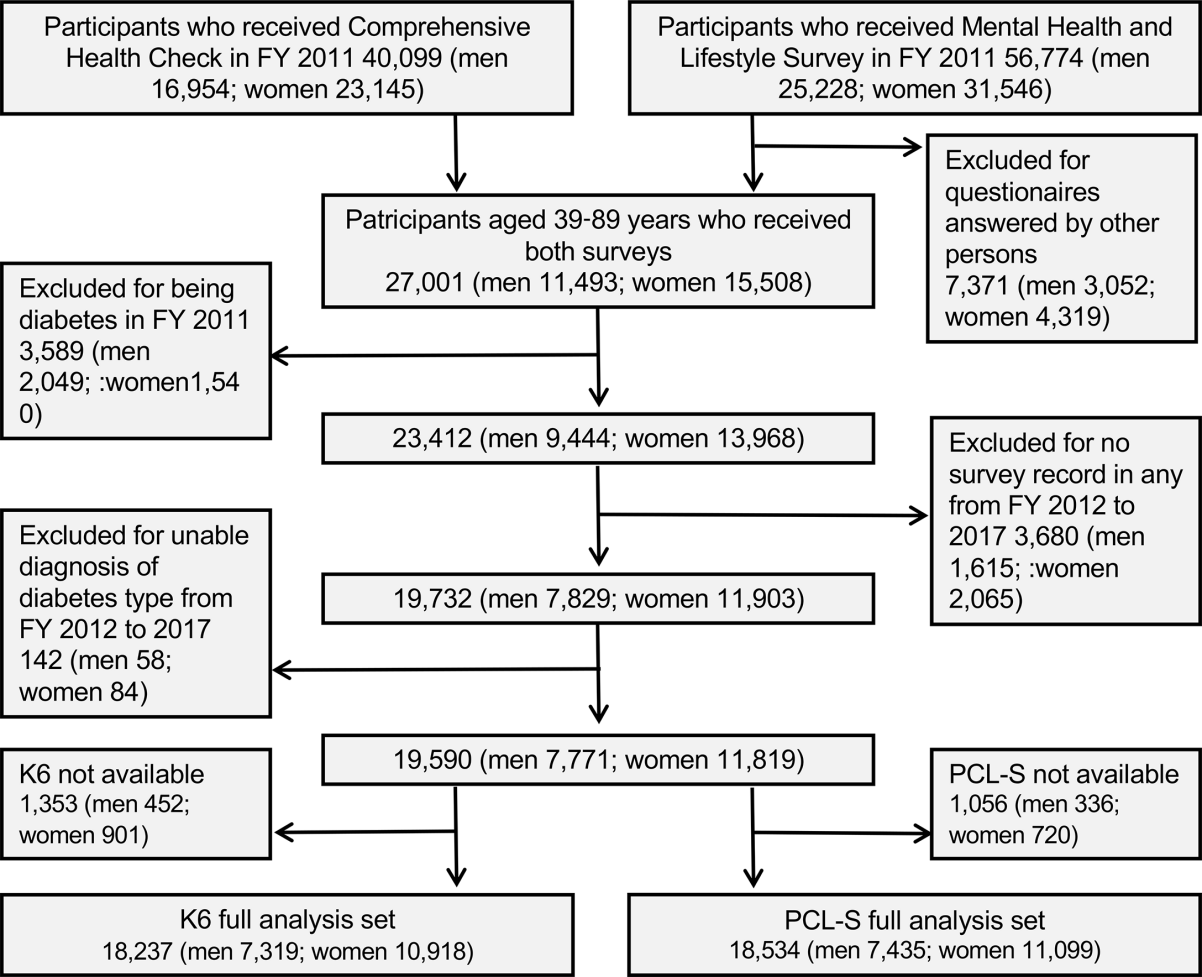
Enrollment flowchart of studied participants in Fukushima Health Management Survey. FY: fiscal year; K6: Kessler 6, PCL-S: PTSD Checklist Stressor-Specific Version.

This study was part of the Fukushima Health Management Survey that targeted 123,314 people aged 40–74 years at the time of the earthquake and was officially registered as being from 13 administrative districts (villages, towns, and cities), which included the evacuation zone (18, 22). The administrative districts included an evacuation zone and a non-evacuation zone. The Fukushima health management survey includes four detailed annual surveys: thyroid ultrasound examination, comprehensive health checks, mental health and lifestyle surveys, and pregnancy and birth surveys (18). Among the participants who underwent a medical health check (n = 40,099) and those who received the mental health survey (n = 56,774) between July 2011 and November 2012, we selected 27,001 participants (men 11,493, women 15,508) who underwent the two surveys (**Figure 1**). After excluding patients with diabetes (n = 3,589), no follow-up examinations (n = 3,680), and missing data for diabetes diagnosis (n = 142), 19,590 participants were included in the full analysis set. The study protocol was approved by the Ethics Review Committee of Fukushima Medical University (#29064), and all participants provided written informed consent.

### Mental health assessment

To assess participants’ mental health status, we used the Kessler 6 scale (K6) (23) and PTSD Checklist—Stressor-Specific Version (PCL-S) (24). The K6 scale used to measure non-specific mental health distress asked participants if they had experienced any of the 6 symptoms during the preceding 30 days: ‘feeling so sad that nothing could cheer you up,’ ‘feeling nervous,’ ‘feeling hopeless,’ ‘feeling restless or fidgety,’ ‘feeling everything was an effort,’ and ‘feeling worthless.’ Each question was scored using a 5-point Likert-type scale from 0 to 4, with higher scores indicating poorer mental health; thus, the total scores ranged from 0 to 24. The Japanese version of K6 has been previously validated (25, 26). Probable depression was defined as a K6 score of ≥ 13 (26). The PCL-S used to measure the traumatic symptoms caused by the Great East Japan Earthquake is a 17-item self-reported measure. We classified participants as having probable PTSD if their PCL-S total score was ≥ 44 (24). The Japanese version of the PCL-S was previously validated by the Fukushima Health Management Survey (27).

### Diabetes- and disaster-related variables

General participant characteristics and diabetes- or disaster-related variables were assessed using self-report questionnaires. Smoking status was classified into three categories: never smoking, former smoking, and current smoking. Drinking status was classified as never drinking, former drinking, current drinking < 40 g/day in men and < 20 g/day in women, or current drinking ≥ 40 g/day in men and ≥ 20 g/day in women. Physical activity was classified into four categories: almost every day, 2–4 times/week, once/week, and almost never.

Participants were grouped into “evacuation” and “no evacuation.” Participants with evacuation were defined as those from the evacuation zone or those from the non-evacuation zone who experienced living arrangements such as evacuation shelters and temporary housing. Post-disaster changes in work situations, including loss of employment and decrease in income, were answered with ‘yes’ or ‘no.’ Post-disaster sleep habits were classified into four categories: satisfied, slightly dissatisfied, quite dissatisfied, and very dissatisfied/have not slept. Educational attainment was divided into elementary school or junior high school (≤ 9 years of education), high school (10–12 years of education), vocational college or junior college (13–15 years of education), and university or graduate school (≥ 16 years of education) as described (28).

The laboratory data obtained from the participants included measurements of aspartate aminotransferase (AST), alanine aminotransferase (ALT), gamma-glutamyl transpeptidase (γ-GT), high-density lipoprotein cholesterol (HDL-C), low-density lipoprotein cholesterol (LDL-C), triglycerides, fasting plasma glucose (FPG), and HbA1c. Diabetes was defined as FPG level ≥ 126 mg/dL, HbA1c level ≥ 6·5%, or self-reported use of antihyperglycemic agents. Hypertension was defined as a systolic blood pressure ≥ 140 mmHg, diastolic blood pressure ≥ 90 mmHg, or self-reported use of antihypertensive agents. Dyslipidemia was defined as an LDL-C level ≥ 140 mg/dL, triglyceride level ≥ 150 mg/dL, HDL cholesterol level < 40 mg/dL, or the use of lipid-lowering agents. Height (in stocking feet) and weight (wearing light clothing) were measured for each participant; BMI was calculated as weight (kg) divided by the square of the height (m^2^); overweight was defined as a BMI ≥ 25 kg/m^2^.

### Statistical analysis

Values are expressed as mean (standard deviation [SD] or confidence interval [95% CI]), number (%), or median (interquartile range [IQR], 25–75%]). Group comparisons were evaluated using Fisher’s exact test for categorical variables and the Mann–Whitney *U* test or Student’s *t*-test for continuous variables. Non-diabetic participants were categorized into K6 < 13 vs. K6 ≥ 13 or PCL-S < 44 vs. PCL-S ≥ 44 among both men and women. Kaplan-Meier survival curves for new-onset diabetes mellitus were constructed, and the probabilities were compared using a log-rank test between groups. Cox proportional hazard models were used to investigate the factors associated with new-onset diabetes mellitus. Factors evaluated included age (year), men (vs women), BMI < 18·5 (vs 18.5-24.9), BMI ≥ 25 (vs 18.5-24.9), hypertension, dyslipidemia, current smoking (vs no current smoking), former and current drinking < 40 g/day ≥ 40 g/day in men,< 20 g/day ≥ 20 g/day in women (vs never drinking), physical activity ≥ 2/week (vs <2/week), evacuation (vs no evacuation), change in work situation (vs no change in work situation), sleep satisfied (vs not dissatisfied), education ≥ 13 years (vs< 13), K6 ≥ 13 (vs < 13), and PCL-S ≥ 44 (vs < 44). Unadjusted, age- and sex-adjusted, and multivariate-adjusted hazard ratios (HR) and 95% CIs were calculated for all, men, and women. To investigate the effects of disaster-related variables on the associations between K6 ≥ 13 and PCL-S ≥ 44 and new-onset type diabetes mellitus, we constructed multivariate Cox proportional hazards models: Model 1: unadjusted; Model 2: Model 1+ age sex, and BMI (3 categories); Model 3: Model 2+ hypertension and dyslipidemia; Model 4: Model 3+ smoking habit, drinking habit, and physical activity; Model 5: Model 4+ evacuation; Model 6: Model 5 + sleep satisfied, Model 7: Model 6 + education ≥ 13 years, and Model 8: Model 7 + change in work situation. All statistical analyses were conducted using SPSS Statistics for Windows (version 23.0, IBM, Armonk, New York, NY, USA). All tests were two-sided, P < 0.05 was considered statistically significant.

## Results

### General characteristics

#### Men vs. women

The baseline characteristics of all men and women are shown in **Table 1**. A total of 19,590 participants showed a mean age of 62·5 (SD 10.8) years and 39·7% were men. Men had a lower median score in the K6 and PCL-S groups. Men were older and had a higher prevalence of BMI ≥ 25, hypertension, currentNew onset diabetes mellitus smoking, and regular exercise. Men had a lower prevalence of sleep dissatisfaction and a comparable rate of evacuation and change in work situation than women. Men had a higher fasting plasma glucose level but a slightly lower HbA1c level (men 5·3% vs. women 5·4%).

**Table 1:** General characteristics of participants at baseline.

					All			Men			Women			All			Men			Women		
	All	Men	Women	P value	K6 < 13	K6 ≥ 13	P value	K6 < 13	K6 ≥ 13	P value	K6 < 13	K6 ≥ 13	P value	PCL-S < 44	PCL-S ≥ 44	P value	PCL-S < 44	PCL-S ≥ 44	P value	PCL-S < 44	PCL-S ≥ 44	P value
Subjects, n (%)	19,590	7771 (39.7)	11819 (60.3)		15,528 (85.1)	2709 (14.9)		6523 (89.1)	796 (10.9)		9005 (82.5)	1913 (17.5)		14364 (77.5)	4170 (22.5)		6064 (81.6)	1371 (18.4)		8300 (74.8)	2799 (25.2)	
Age (years), mean (SD)	62.5 (10.8)	64.1 (10.6)	61.5 (10.8)	<0.001	62.0 (10.7)	62.0 (10.5)	0.996	63.7 (10.6)	62.9 (10.3)	0.052	60.7 (10.6)	61.6 (10.5)	0.001	61.6 (10.7)	63.5 (10.5)	<0.001	63.5 (10.6)	65.0 (10.4)	<0.001	60.3 (10.6)	62.8 (10.4)	<0.001
Men, n (%)					6,523 (42.0)	796 (29.4)	<0.001							6,064 (42.2)	1,371 (32.9)	<0.001						
K6 score, median (Q1-Q3)	5 (2-10)	4 (1-9)	6 (2-11)	<0.001	4 (1-8)	16 (14-19)	<0.001	3 (0-7)	16 (14-19)	<0.001	5 (2-8)	16 (14-19)	<0.001	4 (1-7)	12 (9-16)	<0.001	3 (0-6)	12 (8-16)	<0.001	5 (2-8)	13 (9-17)	<0.001
PCL score, median (Q1-Q3)	30 (22-41)	27 (20-39)	31 (23-43)	<0.001	27 (21-36)	53 (43-64)	<0.001	25 (20-35)	52 (43-63)	<0.001	28 (22-37)	54 (43-64)	<0.001	26 (20-33)	54 (48-62)	<0.001	24 (19-32)	53 (48-62)	<0.001	27 (21-34)	54 (48-63)	<0.001
**New onset diabetes mellitus**																						
Person•years, total	86,608	33,172	53,436		68,973	12,009		28,047	3,355		40,926	8,655		63,849	18,440		26,110	5,767		37,739	12,673	
Folow-up periods (years), mean	4.4	4.3	4.5		4.4	4.4		4.3	4.2		4.5	4.5		4.4	4.4		4.3	4.2		4.5	4.5	
Incidence cases, n	1,699	911	788		1,316	251		738	113		578	138		1,200	401		674	192		526	209	
Incidence rate (/1,000 persons•years)	19.6	27.5	14.7		19.1	20.9		26.3	33.7		14.1	15.9		18.8	21.7		25.8	33.3		13.9	16.5	
**Anthropometry**																						
Systolic blood pressure (mmHg), mean (SD)	131(16.1)	134 (15.5)	129 (16.3)	<0.001	131 (16.1)	130 (15.9)	<0.001	134 (15.5)	133 (15.3)	0.258	129 (16.3)	128 (15.9)	0.122	131 (16.1)	131 (16.1)	0.436	133 (15.4)	134 (15.6)	0.461	129 (16.3)	129 (16.1)	0.017
Diastolic blood pressure (mmHg), mean (SD)	79 (10.2)	81 (9.9)	77 (10.1)	<0.001	79 (10.2)	78 (10.3)	<0.001	81 (10.0)	81 (10.0)	0.644	77 (10.1)	77 (10.2)	0.011	79 (10.2)	78 (10.3)	0.082	81 (9.9)	81 (10.2)	0.980	77 (10.1)	77 (10.2)	0.755
Body weight (kg), mean (SD)	58.5 (10.6)	65.3 (9.8)	54.1 (8.6)	<0.001	58.9 (10.6)	57.5 (10.8)	<0.001	65.5 (9.8)	66.2 (9.9)	0.066	54.2 (8.5)	53.9 (8.9)	0.269	58.9 (10.6)	57.9 (10.6)	<0.001	65.4 (9.7)	65.5 (10.2)	0.787	54.2 (8.5)	54.2 (8.7)	0.985
Body mass index (kg/m^2^), mean (SD)	23.7(3.3)	24.2(3.0)	23.3(3.4)	<0.001	23.6(3.3)	23.6(3.5)	0.604	24.2(3.0)	24.4(3.1)	0.024	23.2(3.4)	23.2(3.6)	0.898	23.6(3.3)	23.8(3.4)	<0.001	24.2(3.0)	24.4(3.1)	0.005	23.2(3.4)	23.5(3.5)	<0.001
Body mass index (kg/m^2^), n (%)				<0.001			0.103			0.211			0.379			0.003			0.005			0.005
Missing	8 (0.0)	2 (0.0)	6 (0.1)		5 (0.0)	3 (0.1)		2 (0.0)	0 (0.0)		3 (0.0)	3 (0.2)		6 (0.0)	2 (0.0)		2 (0.0)	0 (0.0)		4 (0.0)	2 (0.1)	
< 18.5	875 (4.5)	167 (2.1)	708 (6.0)		679 (4.4)	141 (5.2)		142 (2.2)	12 (1.5)		537 (6.0)	129 (6.7)		635 (4.4)	190 (4.6)		125 (2.1)	31 (2.3)		510 (6.1)	159 (5.7)	
18.5 - 25.0	12,510 (63.9)	4,680 (60.2)	7,830 (66.2)		9,966 (64.2)	1,745 (64.4)		3,930 (60.2)	465 (58.4)		6,036 (67.0)	1,280 (66.9		9,277 (64.6)	2,576 (61.8)		3,701 (61.0)	772 (56.3)		5,576 (67.2)	1,804 (64.5)	
≥ 25.0	6,197 (31.6)	2,922 (37.6)	3,275 (27.7)		4,878 (31.4)	820 (30.3)		2,449 (37.5)	319 (40.1)		2,429 (27.0)	501 (26.2)		4,446 (31.0)	1,402 (33.6)		2,236 (36.9)	568 (41.4)		2,210 (26.6)	834 (29.8)	
**Blood measurements**																						
Fasting plasma glucose (mg/dl), mean (SD)	97 (9.6)	99 (9.7)	95 (9.2)	<0.001	97 (9.6)	96 (9.7)	0.012	99 (9.7)	99 (10.1)	0.820	95 (9.1)	95 (9.2)	0.692	96 (9.5)	96 (9.8)	0.652	99 (9.7)	99 (9.9)	0.433	95 (9.1)	95 (9.4)	0.141
HbA1c (%), mean (SD)	5.4 (0.4)	5.3 (0.4)	5.4 (0.3)	0.016	5.3 (0.4)	5.4 (0.4)	0.339	5.3 (0.4)	5.4 (0.4)	0.455	5.4 (0.3)	5.4 (0.4)	0.668	5.3 (0.4)	5.4 (0.4)	0.029	5.3 (0.4)	5.4 (0.4)	0.390	5.3 (0.3)	5.4 (0.4)	0.056
HDL cholesterol (mg/dl), mean (SD)	61 (15.2)	56 (14.4)	64 (14.9)	<0.001	61 (15.3)	61 (15.2)	0.450	56 (14.5)	55 (13.5)	0.079	64 (14.9)	63 (15.2)	0.034	61 (15.3)	60 (15.1)	0.034	56 (14.5)	55 (14.0)	0.002	64 (14.9)	63 (14.8)	<0.001
LDL cholesterol (mg/dl), mean (SD)	127 (32.1)	123 (31.7)	130 (32.1)	<0.001	127 (32.0)	127 (32.7)	0.365	123 (31.7)	124 (32.8)	0.410	130 (32.0)	128 (32.6)	0.002	127 (32.0)	126 (32.1)	0.117	123 (31.8)	123 (31.3)	0.900	130 (31.9)	128 (32.3)	0.001
Tryglicerides (mg/dl), mean (SD)	113 (73.0)	128 (92.3)	103 (54.6)	<0.001	113 (72.9)	114 (80.3)	0.300	128 (90.6)	137 (117.3)	0.019	102 (54.3)	105 (55.7)	0.034	112 (72.8)	117 (78.1)	<0.001	127 (90.6)	134 (105.6)	0.003	101 (53.6)	108 (58.2)	<0.001
**Comorbidities**																						
Hypertension, n (%)	9,956 (50.9)	4,592 (59.1)	5,364 (45.5)	<0.001	7,731 (49.8)	1,363 (50.4)	0.617	3,816 (58.5)	471 (59.2)	0.687	3,915 (43.6)	892 (46.7)	0.013	7,006 (48.8)	2,260 (54.3)	<0.001	3,497 (57.7)	862 (62.9)	<0.001	3,509 (42.4)	1,398 (50.0)	<0.001
Dyslipidemia, n (%)	11,115 (56.8)	4,275 (55.0)	6,840 (58.0)	<0.001	8,780 (56.6)	1,558 (57.6)	0.359	3,603 (55.3)	445 (55.9)	0.737	5,177 (57.6)	1,113 (58.2)	0.591	8,071 (56.2)	2,453 (58.9)	0.002	3,355 (55.4)	760 (55.5)	0.940	4,716 (56.9)	1,693 (60.6)	<0.001
**Psychosocial factors**																						
Smoking habit, n (%)				<0.001			<0.001			0.107			0.016			<0.001			0.087			0.030
Missing	615 (3.1)	92 (1.2)	523 (4.4)		336 (2.2)	107 (3.9)		54 (0.8)	11 (1.4)		282 (3.1)	96 (5.0)		289 (2.0)	171 (4.1)		49 (0.8)	26 (1.9)		240 (2.9)	145 (5.2)	
Never smoking	12,037 (61.4)	2,089 (26.9)	9,948 (84.2)		9,440 (60.8)	1,787 (66.0)		1,742 (26.7)	226 (28.4)		7,698 (85.5)	1,561 (81.6)		8,715 (60.7)	2,700 (64.7)		1,603 (26.4)	398 (29.0)		7,112 (85.7)	2,302 (82.2)	
Fomer smoking	4,380 (22.4)	3,713 (47.8)	667 (5.6)		3,656 (23.5)	471 (17.4)		3,150 (48.3)	351 (44.1)		506 (5.6)	120 (6.3)		3,394 (23.6)	781 (18.7)		2,915 (48.1)	621 (45.3)		479 (5.8)	160 (5.7)	
Current smoking	2,558 (13.1)	1,877 (24.2)	681 (5.8)		2,096 (13.5)	344 (12.7)		1,577 (24.2)	208 (26.1)		519 (5.8)	136 (7.1)		1,966 (13.7)	518 (12.4)		1,497 (24.7)	326 (23.8)		469 (5.7)	192 (6.9)	
Drinking habit, n (%)				<0.001			<0.001			<0.001			<0.001			<0.001			<0.001			0.009
Missing	410 (2.1)	50 (0.6)	360 (3.0)		198 (1.3)	87 (3.2)		31 (0.5)	3 (0.4)		167 (1.9)	84 (4.4)		179 (1.2)	112 (2.7)		30 (0.5)	9 (0.7)		149 (1.8)	103 (3.7)	
Never drinking	9,928 (50.7)	1,832 (23.6)	8,096 (68.5)		7,730 (49.8)	1,495 (55.2)		1,510 (23.1)	206 (25.9)		6,220 (69.1)	1,289 (67.4)		7,122 (49.6)	2,255 (54.1)		1,414 (23.3)	335 (24.4)		5,708 (68.8)	1,920 (68.6)	
Fomer drinking	504 (2.6)	386 (5.0)	118 (1.0)		371 (2.4)	84 (3.1)		292 (4.5)	56 (7.0)		79 (0.9)	28 (1.5)		330 (2.3)	134 (3.2)		253 (4.2)	103 (7.5)		77 (0.9)	31 (1.1)	
Current drinking: Men < 40, Women < 20 g/day	6,657(34.0)	3,934(50.6)	2,723(23.0)		5,527(35.6)	751(27.7)		3,368(51.6)	347(43.6)		2,159(24.0)	404(21.1)		5,127(35.7)	1,243(29.8)		3,120(51.5)	646(47.1)		2,007(24.2)	597(21.3)	
Current drinking: Men ≥ 40, Women 20 > g/day	2,091(10.7)	1,569(20.2)	522(4.4)		1,702(11.0)	292(10.8)		1,322(20.3)	184(23.1)		380(4.2)	108(5.6)		1,606(11.2)	426(10.2)		1,247(20.6)	278(20.3)		359(4.3)	148(5.3)	
Physical activity, n (%)				<0.001			<0.001			<0.001			0.130			<0.001			0.062			<0.001
Missing	472 (2.4)	172 (2.2)	300 (2.5)		263 (1.7)	52 (1.9)		100 (1.5)	15 (1.9)		163 (1.8)	37 (1.9)		244 (1.7)	107 (2.6)		97 (1.6)	35 (2.6)		147 (1.8)	72 (2.6)	
Almost every day	3,340 (17.0)	1,654 (21.3)	1,686 (14.3)		2,699 (17.4)	378 (14.0)		1,401 (21.5)	136 (17.1)		1,298 (14.4)	242 (12.7)		2,444 (17.0)	674 (16.2)		1,282 (21.1)	284 (20.7)		1,162 (14.0)	390 (13.9)	
2-4 times/week	4,803 (24.5)	1,862 (24.0)	2,941 (24.9)		3,760 (24.2)	655 (24.2)		1,563 (24.0)	185 (23.2)		2,197 (24.4)	470 (24.6)		3,389 (23.6)	1,122 (26.9)		1,421 (23.4)	363 (26.5)		1,968 (23.7)	759 (27.1)	
1 times/week	2,895 (14.8)	1,106 (14.2)	1,789 (15.1)		2,315 (14.9)	377 (13.9)		949 (14.5)	100 (12.6)		1,366 (15.2)	277 (14.5)		2,120 (14.8)	622 (14.9)		876 (14.4)	192 (14.0)		1,244 (15.0)	430 (15.4)	
Almost never	8,080 (41.2)	2,977 (38.3)	5,103 (43.2)		6,491 (41.8)	1,247 (46.0)		2,510 (38.5)	360 (45.2)		3,981 (44.2)	887 (46.4)		6,167 (42.9)	1,645 (39.4)		2,388 (39.4)	497 (36.3)		3,779 (45.5)	1,148 (41.0)	
Evacuation, n (%)	10,786 (55.1)	4,223 (54.3)	6,563 (55.5)	0.103	8,169 (52.6)	1,834 (67.7)	<0.001	3,404 (52.2)	555 (69.7)	<0.001	4,765 (52.9)	1,279 (66.9)	<0.001	7,447 (51.8)	2,734 (65.6)	<0.001	3,106 (51.2)	916 (66.8)	<0.001	4,341 (52.3)	1,818 (65.0)	<0.001
Change in work situation, n (%)	10,384 (56.6)	4,255 (57.2)	6,129 (56.2)	0.191	7,928 (53.7)	1,796 (71.0)	<0.001	3,436 (54.5)	584 (75.5)	<0.001	4,492 (53.1)	1,212 (68.9)	<0.001	7,172 (52.3)	2,700 (69.7)	<0.001	3,120 (53.1)	959 (73.7)	<0.001	4,052 (51.8)	1,741 (67.7)	<0.001
Sleep satisfaction, n (%)				<0.001			<0.001			<0.001			<0.001			<0.001			<0.001			<0.001
Missing	3,634 (18.6)	1,418 (18.2)	2,216 (18.7)		2,739 (17.6)	483 (17.8)		1,143 (17.5)	140 (17.6)		1,596 (17.7)	343 (17.9)		2,433 (16.9)	808 (19.4)		1,030 (17.0)	266 (19.4)		1,403 (16.9)	542 (19.4)	
Satisfied	5,345 (27.3)	2,679 (34.5)	2,666 (22.6)		4,847 (31.2)	214 (7.9)		2,469 (37.9)	85 (10.7)		2,378 (26.4)	129 (6.7)		4,702 (32.7)	424 (10.2)		2,390 (39.4)	194 (14.2)		2,312 (27.9)	230 (8.2)	
Slightly dissatisfied	7,380 (37.7)	2,659 (34.2)	4,721 (39.9)		6,077 (39.1)	849 (31.3)		2,275 (34.9)	249 (31.3)		3,802 (42.2)	600 (31.4)		5,691 (39.6)	1,384 (33.2)		2,128 (35.1)	444 (32.4)		3,563 (42.9)	940 (33.6)	
Quite dissatisfied	2,523 (12.9)	822 (10.6)	1,701 (14.4)		1,586 (10.2)	803 (29.6)		554 (8.5)	222 (27.9)		1,032 (11.5)	581 (30.4)		1,343 (9.3)	1,092 (26.2)		459 (7.6)	339 (24.7)		884 (10.7)	753 (26.9)	
Very dissatisfied	708 (3.6)	193 (2.5)	515 (4.4)		279 (1.8)	360 (13.3)		82 (1.3)	100 (12.6)		197 (2.2)	260 (13.6)		195 (1.4)	462 (11.1)		57 (0.9)	128 (9.3)		138 (1.7)	334 (11.9)	
Education, n (%)				<0.001			<0.001			0.012			<0.001			<0.001			<0.001			<0.001
Missing	728 (3.7)	265 (3.4)	463 (3.9)		476(3.1)	88(3.2)		187(2.9)	26(3.3)		289(3.2)	62(3.2)		398(2.8)	165(4.0)		162(2.7)	57(4.2)		236(2.8)	108(3.9)	
≤ 9 years	5,211 (26.6)	2,334 (30.0)	2,877 (24.3)		3,866(24.9)	738(27.2)		1,854(28.4)	261(32.8)		2,012(22.3)	477(24.9)		3,542(24.7)	1,213(29.1)		1,725(28.4)	448(32.7)		1,817(21.9)	765(27.3)	
≤ 12 years	9,644 (49.2)	3,709(47.7)	5,935 (50.2)		7,763(50.0)	1,385(51.1)		3,170(48.6)	380(47.7)		4,593(51.0)	1,005(52.5)		7,235(50.4)	2,051(49.2)		2,947(48.6)	652(47.6)		4,288(51.7)	1,399(50.0)	
13-15 years	2,803 (14.3)	649 (8.4)	2,154 (18.2)		2,354(15.2)	375(13.8)		571(8.8)	61(7.7)		1,783(19.8)	314(16.4)		2,158(15.0)	576(13.8)		524(8.6)	113(8.2)		1,634(19.7)	463(16.5)	
≥ 16 years	1,204 (6.1)	814 (10.5)	390 (3.3)		1,069(6.9)	123(4.5)		741(11.4)	68(8.5)		328(3.6)	55(2.9)		1,031(7.2)	165(4.0)		706(11.6)	101(7.4)		325(3.9)	64(2.3)	
≥ 13 years	4,007 (20.5)	1,463 (18.8)	2,544 (21.5)	<0.001	3,423(22.0)	498(18.4)	<0.001	1,312(20.1)	129(16.2)	0.009	2,111(23.4)	369(19.3)	<0.001	3,189(22.2)	741(17.8)	<0.001	1,230(20.3)	214(15.6)	<0.001	1,959(23.6)	527(18.8)	<0.001

#### K6 < 13 vs. K6 ≥ 13

In all participants, the mean ages were comparable between K6 < 13 vs. K6 ≥ 13, and men were less in K6 ≥ 13 (42·0% vs. 29.4%). In men, the mean age was slightly lower in K6 ≥ 13, and BMI ≥ 25 was comparable between K6 < 13 and K6 ≥ 13. In women, the mean age was slightly higher in K6 ≥ 13, and BMI ≥ 25 was comparable. In both men and women, the prevalence of evacuation, change in work situation and sleep dissatisfied were higher in K6 ≥ 13.

#### PCL-S < 44 vs. PCL-S ≥ 44

In all patients, the mean age was older, and men were less in PCL-S ≥ 44. In men, the men age was older in PCL-S ≥ 44, and BMI ≥ 25 kg/m^2^ was comparable between PCL-S < 44 and PCL-S ≥ 44. In both men and women, hypertension was higher in PCL-S ≥ 44. Changes in work situation and sleep dissatisfaction were higher in the PCL-S ≥ 44.

### New-onset diabetes mellitus

After seven years of follow-up and a mean follow-up time of 4.4 years (86,609 person-years at risk), 1,699 new cases of type 2 diabetes were identified among 19,590 non-diabetic participants in FY 2011. The incidence of type 2 diabetes was 19·6 (/1,000 person-years) for all, 27·5 for men, and 14·7 for women (**Table 1**).

**Figure 2:**
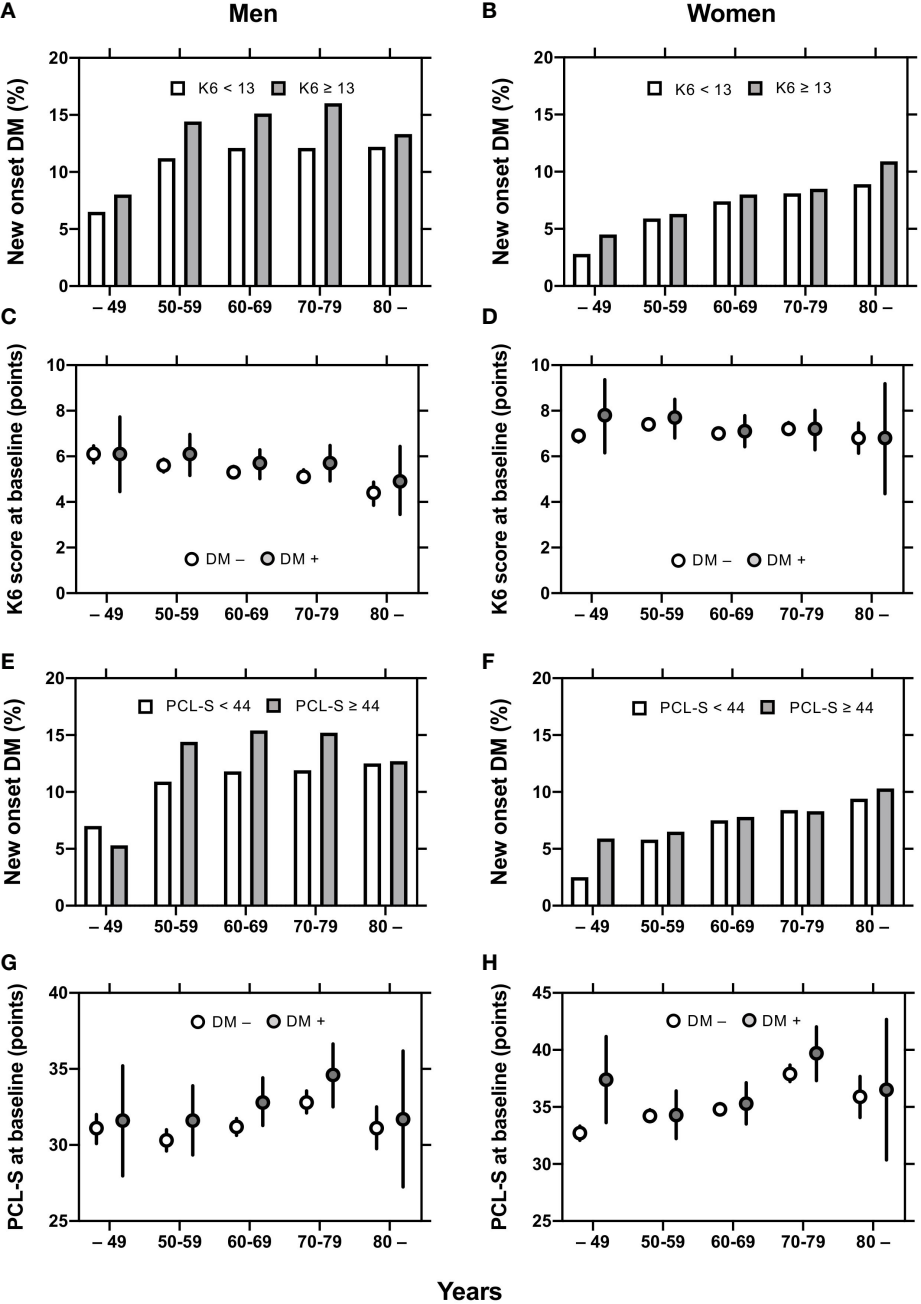
Mean K6 and PCL-S and incidence of new-onset diabetes in men and women. Diabetes incidence by age groups are shown in K6 < 13 (open columns) and K6 ≥ 13 (closed columns) participants **(A, B)** and in PCL-S < 44 (open columns) and PCL-S < 44 (closed columns) participants **(E, F)**. Mean values ± 95% confidence intervals [95% CI] of K6 **(C, D)** and PCL-S **(G, H)** by age groups are shown in participant with (closed circles) or without (open circles) new-onset diabetes mellitus. K6: Kessler 6, PCL-S: PTSD Checklist Stressor-Specific Version; DM: diabetes mellitus.

In men, diabetes incidence by age group was larger in K6 ≥ 13 than in K6 < 13 (**Figure 2A**) and in PCL-S ≥ 44 than in PCL-S < 44 (**Figure 2E**). In women, however, the incidence of diabetes was comparable between the K6 and PCL-S < 44 dichotomies (**Figures 2B, F**). Meanwhile, the mean values of K6 and PCL-S were comparable in FY 2011 between participants with or without new-onset diabetes mellitus, but 95% confidence intervals were larger in participants with new-onset diabetes mellitus in men and women (**Figures 2C, D, G, H**).

### Kaplan–Meier survival curves for new-onset diabetes

**Figure 3:**
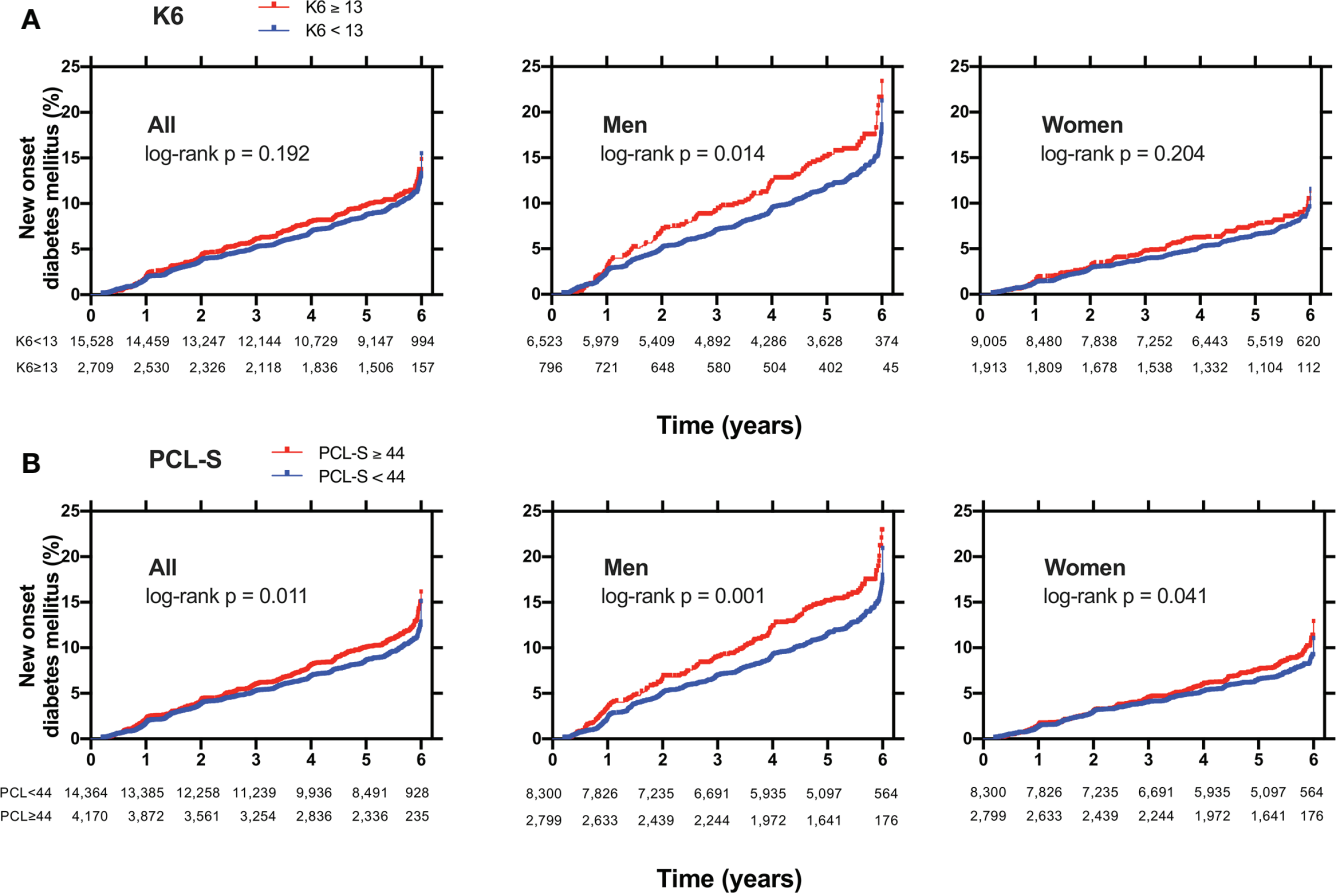
Kaplan Meier curves for new-onset diabetes mellitus in **(A)** participants with K6 < 13 (blue lines) or with K6 ≥ 13 (red lines) or in **(B)** participants with PCL-S < 44 (blue lines) or with PCL-S ≥ 44 (red lines). Non-diabetic participants were plotted for new-onset diabetes mellitus in all, men, and women. K6: Kessler 6, PCL-S: PTSD Checklist Stressor-Specific Version; DM: diabetes mellitus; P: p values calculated by log-rank test.

The Kaplan–Meier curves for new-onset diabetes are shown in **Figure 3**. The log-rank test indicated a significant difference between K6 ≥ 13 and K6 < 13 in men (p = 0·014) but not in all and women (**Figure 3A**). There were significant differences between PCL-S ≥ 44 and PCL-S < 44 in all (p = 0·011), men (p = 0·001), and women (p = 0·041) (**Figure 3B**).

### Cox proportional hazards model for new-onset diabetes mellitus

The univariate and multivariate Cox proportional hazards models for new-onset diabetes are shown in **Table 2**.

Table 2Factors associated with new-onset diabetes mellitus.

**Table:** 

A. K6 full analysis set																												
		All (n = 18,237)									Men (n = 7,319)									Women (n = 10,918)								
		Unadjusted			Age- and sex-adjusted			Multivariate-adjusted			Unadjusted			Age- and sex-adjusted			Multivariate-adjusted			Unadjusted			Age- and sex-adjusted			Multivariate-adjusted		
Factors	Reference	HR	95%CI		HR	95%CI		HR	95%CI		HR	95%CI		HR			HR	95%CI		HR	95%CI		HR	95%CI		HR	95%CI	
Age (year)	Per year	1.03	1.02	1.03	1.02	1.02	1.03	1.01	1.01	1.02	1.01	1.01	1.02	1.01	1.01	1.02	1.01	1.00	1.02	1.02	1.03	1.04	1.03	1.02	1.04	1.01	1.003	1.02
Men	Women	1.90	1.72	2.09	1.78	1.61	1.97	1.64	1.45	1.85																		
Body mass index < 18.5	18.5 - 25.0	0.52	0.36	0.77	0.61	0.41	0.89	0.70	0.48	1.03	0.84	0.45	1.57	0.84	0.45	1.57	0.97	0.51	1.82	0.52	0.32	0.85	0.55	0.34	0.89	0.65	0.40	1.06
Body mass index ≥ 25.0	18.5 - 25.0	2.33	2.11	2.58	2.18	1.97	2.41	1.86	1.68	2.07	1.94	1.70	2.23	1.96	1.71	2.25	1.73	1.50	1.99	2.54	2.19	2.94	2.45	2.11	2.84	2.05	1.76	2.39
Hypertention	No hypertention	2.40	2.15	2.67	2.06	1.84	2.31	1.74	1.55	1.96	1.86	1.60	2.16	1.79	1.54	2.09	1.61	1.37	1.88	2.67	2.28	3.11	2.36	2.00	2.79	1.87	1.58	2.22
Dyslipidemia	No dyslipidemia	1.65	1.49	1.84	1.64	1.47	1.82	1.42	1.27	1.58	1.44	1.25	1.65	1.46	1.27	1.68	1.31	1.13	1.51	2.08	1.76	2.46	1.87	1.58	2.21	1.57	1.33	1.87
Current smoking	No current smoking	1.16	1.01	1.34	1.03	0.89	1.20	1.10	0.95	1.28	0.95	0.81	1.12	1.03	0.87	1.21	1.10	0.93	1.30	0.73	0.50	1.05	0.90	0.62	1.31	0.98	0.67	1.43
Fomer drinking	Never drinking	1.71	1.30	2.24	1.10	0.83	1.45	1.15	0.87	1.51	1.17	0.85	1.60	1.11	0.81	1.53	1.13	0.82	1.56	1.40	0.75	2.61	1.51	0.80	2.82	1.28	0.68	2.40
Current drinking	Never drinking	1.15	1.03	1.28	0.89	0.79	1.01	0.92	0.82	1.04	0.93	0.78	1.10	0.93	0.79	1.10	0.93	0.79	1.11	0.81	0.67	0.97	0.89	0.74	1.08	0.96	0.79	1.15
Current drinking: Men ≥ 40, Women 20 > g/day	Never drinking	1.32	1.13	1.54	0.97	0.82	1.15	0.95	0.80	1.14	1.01	0.83	1.23	1.05	0.86	1.29	0.992	0.81	1.22	0.50	0.31	0.81	0.62	0.38	1.02	0.69	0.42	1.13
Physical activity ≥ 2/week	< 2/week	1.16	1.05	1.28	0.95	0.85	1.06	0.98	0.88	1.09	1.06	0.93	1.22	0.96	0.83	1.11	0.98	0.84	1.13	1.18	1.02	1.37	0.95	0.82	1.11	0.99	0.85	1.16
Evacuation	No evacuation	1.17	1.05	1.29	1.21	1.09	1.34	1.14	1.03	1.27	1.23	1.07	1.41	1.25	1.09	1.43	1.16	1.000	1.34	1.12	0.96	1.30	1.16	1.000	1.35	1.12	0.96	1.31
Change in work situation	No change of job	1.07	0.97	1.19	1.15	1.04	1.28	1.05	0.94	1.17	1.18	1.03	1.36	1.25	1.08	1.44	1.15	0.99	1.33	0.94	0.80	1.09	1.04	0.89	1.21	0.95	0.81	1.11
Sleep satisfied	Sleep dissatisfied	0.91	0.80	1.04	0.82	0.72	0.94	0.89	0.77	1.02	0.996	0.81	1.22	0.97	0.79	1.18	1.08	0.87	1.34	0.74	0.62	0.89	0.72	0.60	0.86	0.74	0.61	0.90
Education ≥ 13 years	< 13	0.75	0.65	0.85	0.85	0.74	0.97	0.89	0.78	1.02	0.90	0.76	1.07	0.95	0.80	1.13	0.98	0.82	1.17	0.64	0.52	0.78	0.74	0.61	0.91	0.79	0.64	0.97
K6 ≥ 13	< 13	1.09	0.96	1.25	1.19	1.04	1.37	1.09	0.95	1.26	1.28	1.05	1.56	1.30	1.06	1.58	1.23	1.000	1.52	1.13	0.94	1.36	1.10	0.91	1.32	0.990	0.81	1.21

**Table:** 

B. PCL-S full analysis set																						
		All (n = 18,534)									Men (n = 7,435)									Women (n = 11,099)								
		Unadjusted			Age- and sex-adjusted			Multivariate-adjusted			Unadjusted			Age- and sex-adjusted			Multivariate-adjusted			Unadjusted			Age- and sex-adjusted			Multivariate-adjusted		
Factors	Reference	HR	95%CI		HR	95%CI		HR	95%CI		HR	95%CI		HR			HR	95%CI		HR	95%CI		HR	95%CI		HR	95%CI	
Age (year)	Per year	1.03	1.02	1.03	1.02	1.02	1.03	1.01	1.01	1.02	1.01	1.01	1.02	1.01	1.01	1.02	1.01	1.00	1.02	1.03	1.03	1.04	1.03	1.03	1.04	1.01	1.00	1.02
Men	Women	1.88	1.71	2.08	1.76	1.59	1.94	1.64	1.45	1.85																		
Body mass index < 18.5	18.5 - 25	0.53	0.37	0.78	0.62	0.43	0.91	0.72	0.49	1.05	0.90	0.50	1.65	0.90	0.49	1.63	1.04	0.57	1.90	0.52	0.32	0.84	0.55	0.34	0.89	0.65	0.40	1.05
Body mass index ≥ 25	18.5 - 25	2.31	2.09	2.55	2.17	1.96	2.39	1.85	1.67	2.05	1.90	1.66	2.17	1.92	1.68	2.20	1.69	1.47	1.94	2.56	2.21	2.97	2.46	2.12	2.85	2.07	1.78	2.41
Hypertention	No hypertention	2.38	2.14	2.65	2.04	1.82	2.28	1.72	1.54	1.93	1.85	1.59	2.14	1.77	1.52	2.06	1.59	1.36	1.86	2.66	2.28	3.10	2.33	1.97	2.74	1.84	1.55	2.17
Dyslipidemia	No dyslipidemia	1.63	1.47	1.81	1.62	1.45	1.80	1.40	1.26	1.56	1.43	1.24	1.64	1.45	1.26	1.66	1.31	1.14	1.51	2.04	1.73	2.40	1.81	1.54	2.14	1.54	1.30	1.82
Current smoking	No current smoking	1.17	1.02	1.35	1.05	0.91	1.22	1.11	0.96	1.29	0.97	0.83	1.14	1.05	0.90	1.24	1.12	0.95	1.33	0.68	0.47	0.998	0.86	0.58	1.25	0.93	0.63	1.37
Fomer drinking	Never drinking	1.64	1.25	2.15	1.06	0.80	1.40	1.11	0.84	1.47	1.17	0.85	1.61	1.12	0.81	1.54	1.13	0.82	1.56	1.18	0.61	2.29	1.28	0.66	2.48	1.13	0.58	2.18
Current drinking	Never drinking	1.14	1.02	1.27	0.89	0.79	1.003	0.92	0.81	1.04	0.94	0.80	1.11	0.95	0.80	1.12	0.95	0.80	1.12	0.79	0.65	0.94	0.87	0.73	1.05	0.93	0.78	1.12
Current drinking: Men ≥ 40, Women 20 > g/day	Never drinking	1.31	1.12	1.52	0.97	0.82	1.15	0.95	0.80	1.13	1.02	0.84	1.25	1.07	0.88	1.31	1.01	0.83	1.24	0.50	0.31	0.80	0.62	0.39	1.000	0.68	0.42	1.10
Physical activity ≥ 2/week	< 2/week	1.15	1.04	1.27	0.93	0.84	1.03	0.96	0.86	1.06	1.06	0.93	1.22	0.95	0.83	1.10	0.97	0.84	1.12	1.15	0.99	1.33	0.92	0.78	1.07	0.95	0.82	1.11
Evacuation	No evacuation	1.16	1.05	1.28	1.20	1.09	1.33	1.13	1.02	1.26	1.22	1.06	1.40	1.24	1.08	1.42	1.15	0.99	1.32	1.12	0.96	1.29	1.16	1.00	1.34	1.12	0.96	1.30
Change in work situation	No change of job	1.07	0.96	1.18	1.14	1.03	1.27	1.05	0.94	1.16	1.17	1.02	1.34	1.24	1.07	1.42	1.12	0.97	1.30	0.93	0.80	1.09	1.04	0.89	1.21	0.96	0.82	1.13
Sleep satisfied	Sleep dissatisfied	0.91	0.80	1.04	0.82	0.72	0.94	0.89	0.77	1.02	0.997	0.82	1.22	0.97	0.79	1.18	1.10	0.89	1.36	0.74	0.62	0.89	0.72	0.61	0.87	0.73	0.61	0.89
Education ≥ 13 years	< 13	0.72	0.63	0.83	0.82	0.72	0.94	0.86	0.75	0.99	0.88	0.74	1.05	0.93	0.78	1.11	0.97	0.81	1.16	0.61	0.50	0.75	0.71	0.58	0.88	0.76	0.62	0.94
PCL-S ≥ 44	< 44	1.16	1.03	1.30	1.19	1.06	1.33	1.06	0.94	1.20	1.30	1.11	1.53	1.28	1.09	1.50	1.20	1.01	1.43	1.18	1.01	1.39	1.09	0.93	1.29	0.95	0.80	1.13

HR, hazard ratio; CI, confidential intervals.

In all K6 full analysis sets (**Table 2A**), multivariate-adjusted HR associated with new-onset diabetes was significant in age, men, BMI ≥ 25, hypertension, and dyslipidemia. In men, the multivariate-adjusted HR of age BMI ≥ 25, hypertension, and dyslipidemia were associated with new-onset diabetes. The multivariate-adjusted HR was significant in evacuation and K6 ≥ 13. The age- and sex-adjusted HR, but not multivariate-adjusted HR, was significant in change in work situation. In women, the multivariate-adjusted HR was significantly associated with age BMI ≥ 25, hypertension, and dyslipidemia, but not with evacuation, change in work situation, and K6 ≥ 13. HR in current smoking, current drinking, physical activity ≥ 2/week, and sleep satisfaction were not significantly associated with new-onset diabetes in men and women. In contrast, the multivariate-adjusted HR of education ≥ 13 years and sleep dissatisfied were not significant in men but were significantly low in women.

In all PCL-S full analysis sets (**Table 2B**), multivariate-adjusted factors associated with new-onset diabetes were age male sex, BMI ≥ 25, hypertension, dyslipidemia, and evacuation, but not PCL-S ≥ 44. In men, the multivariate-adjusted factors were age BMI ≥ 25, hypertension, dyslipidemia, and PCL-S ≥ 44. In women, multivariate-adjusted factors were age BMI ≥ 25, hypertension, and dyslipidemia but not PCL-S ≥ 44. The multivariate-adjusted HR of education ≥ 13 years and sleep dissatisfied were also significantly low in women.

Next, we evaluated the effects of disaster-related variables on the relationship between K6 ≥ 13, PCL-S ≥44, and new-onset type diabetes mellitus using univariate and multivariate Cox proportional hazards models (**Table 3**).

**Table 3:** Hazard ratio of K6 ≥ 13 or PCL-S ≥44 for new onset diabetes mellitus.

					K6 ≥ 13									PCL-S ≥ 44					
		All (n= 18,237)			Men (n=7,319)			Women (n=10,918)			All (n= 18,534)			Men (n=7,435)			Women (n=11,099)		
	Factors	HR	95%CI			95%CI		HR	95%CI		HR	95%CI		HR	95%CI		HR	95%CI	
Model 1:	Unadjusted	1.09	0.96	1.25	1.28	1.05	1.56	1.13	0.94	1.36	1.16	1.03	1.30	1.30	1.11	1.53	1.18	1.01	1.39
Model 2:	+ Age sex and body mass index (3 categories)	1.19	1.04	1.36	1.27	1.04	1.55	1.11	0.92	1.34	1.15	1.03	1.29	1.24	1.06	1.46	1.06	0.90	1.25
Model 3:	+ Hypertension and dyslipidemia	1.18	1.03	1.35	1.28	1.05	1.56	1.10	0.91	1.32	1.14	1.02	1.28	1.24	1.05	1.45	1.05	0.89	1.23
Model 4:	+ Smoking habit, drinking habit, and physical activity	1.17	1.02	1.34	1.27	1.04	1.55	1.10	0.91	1.32	1.13	1.01	1.27	1.23	1.05	1.44	1.05	0.89	1.23
Model 5:	+ Evacuation	1.14	1.00	1.31	1.23	1.01	1.50	1.08	0.90	1.30	1.11	0.99	1.24	1.20	1.02	1.41	1.03	0.88	1.21
Model 6:	+ Sleep satisfied	1.10	0.95	1.27	1.26	1.02	1.55	0.99	0.81	1.20	1.07	0.95	1.21	1.22	1.03	1.45	0.95	0.80	1.13
Model 7:	+ Education ≥ 13 years	1.10	0.95	1.27	1.26	1.02	1.55	0.98	0.81	1.20	1.07	0.95	1.21	1.22	1.03	1.45	0.94	0.80	1.12
Model 8:	+ Change in work situation	1.09	0.95	1.26	1.23	1.00	1.52	0.99	0.81	1.21	1.06	0.94	1.20	1.20	1.01	1.43	0.95	0.80	1.13

In men, the HR of K6 ≥ 13 remained significant after correcting for age and BMI in three categories (Model 2), hypertension and dyslipidemia (Model 3), smoking habit, drinking habit, physical activity (Model 4), evacuation (Model 5), sleep satisfied (Model 6), education ≥ 13 years (Model 7), and change in work situation (Model 8). In women, K6 ≥ 13 was not a significant factor in the unadjusted or multivariate-adjusted models.

In men, PCL-S ≥ 44 showed significant HRs after correction for all variables, including age BMI, hypertension, dyslipidemia, smoking habit, drinking habit, physical activity, evacuation, sleep satisfied, education ≥ 13 years, and change in work situation. However, in women, the adjusted PCL-S score ≥ 44 was not statistically significant in the multivariate-adjusted models.

## Discussion

This study evaluated the 7-year longitudinal impact of probable depression and probable PTSD on new-onset diabetes mellitus among Fukushima Health Management Survey participants who were survivors of the Great East Japan Earthquake. Two major findings were obtained in this study. First, among all participants, PCL-S ≥ 44 and K6 ≥ 13 were associated with the onset of type 2 diabetes mellitus (**Table 3**). Both K6 ≥ 13 and PCL-S ≥ 44 remained significant in the Cox proportional hazards model after multivariate adjustment for age, male sex, BMI ≥ 25, hypertension, dyslipidemia, smoking habit, drinking habit, physical activity, and evacuation but not after correction for sleep satisfied, education, and change in work situation (**Table 3**). Second, there was a sex difference in the associations between probable depression and probable PTSD on new-onset diabetes mellitus. The multivariate-adjusted Cox model indicated that K6 ≥ 13 and PCL-S ≥ 44 were determinants of new-onset diabetes mellitus in men, independent of evacuation, sleep satisfied, education, and change in work situation. Our results suggest that the post-disaster burden of probable depression and probable PTSD is causally related to new-onset diabetes in men but not in women.

### Association between probable depression and probable PTSD and new-onset diabetes mellitus

It has been implicated that both psychological distress (1–3) and PTSD (8) have causal effects on developing new diabetes mellitus. To our knowledge, however, the effects of psychological distress and PTSD, which are different responses to psychological stress, have never been compared with respect to the onset of diabetes. The current study found that PTSD-L ≥ 44 and K 6 ≥ 13 were both associated with the onset of type 2 diabetes mellitus (**Table 3**).

Previous reports have indicated that depression (4), but not general stress (5) and work stress (6, 7), is an independent risk factor for type 2 diabetes mellitus. Mezuk et al. reported that relative risk for new-onset diabetes associated with baseline depression was 1·60 (1·37–1·88) in the pooled analysis from 13 prospective studies (4). However, no significant association was found between work-related stress and the risk for type 2 diabetes based on a meta-analysis of seven prospective cohort studies (relative risk 0·94 [95% confidence interval 0.72–1.23]) (5).

It remains unclear whether PTSD is associated with a higher risk of developing type 2 diabetes mellitus (8). Vancampfort et al. demonstrated that the relative risk for type diabetes mellitus is 1·49 (95% CI 1·17–1·89, p = 0.001) (8). Three longitudinal case-control studies have been published until now (8). Miller–Archie et al. found a significant association between PTSD and diabetes in a logistic model (multivariate-adjusted odds ratio [AOR]1·28, 95% CI 1·14–1·44) in World Trade Center (WTC) survivors (n = 36,899) up to 11 years after the attack in 2001 (16). Pietrzak et al. reported that PTSD due to lifetime trauma exposures showed an AOR of 1·3 (1·07–1·52) for the diagnosis of diabetes mellitus in American adults (29). Roberts et al. showed that PTSD symptoms were dose-dependent with T2D incidence in a US longitudinal cohort of women (14). However, the authors equally acknowledged the limitations of self-reported diabetes diagnoses (8). This study is the first to demonstrate the relationship between PTSD and the onset of diabetes in a large cohort using a solid definition of diabetes (plasma glucose and HbA1c).

Overall, participants indicated that multivariate-adjusted HR of PCL-S ≥ 44 remained significant after correcting for age male sex, BMI ≥ 25, hypertension, dyslipidemia, smoking habit, drinking habit, and physical activity (**Table 3**), in agreement with the above studies (14, 16, 29). We obtained a new finding that the significance of HR disappeared in the Cox proportional hazards model with correction for covariates of evacuation, sleep dissatisfied, education, and change in work situation. This finding implies that these covariates underlie the cause-and-effect relationship between PTSD and new-onset diabetes. In our previous study, the the evacuation was a risk factor for a 4-year onset of diabetes among survivors of the Great East Japan Earthquake, which is consistent with the results of the current study (30). As changes in the work situation (31) and sleep disorders (32, 33) are considered to be associated with new-onset diabetes independently of PTSD, the cause-and-effect relationship between PTSD and these covariates should be carefully interpreted.

### Gender difference in the relationship between probable depression and probable PTSD and new-onset diabetes mellitus

Previous studies have reported that depression (1–3, 34, 35) and PTSD (8, 14, 36, 37) are factors in the development of diabetes mellitus in both men and women, but there are also reports of gender differences (9, 38).

Eriksson et al. found that the AOR for new-onset diabetes was 2·2 (95%CI 1·2–4·1) in men and 0·5 (0·2–1·2) in women in an 8–10 years cohort study comprised Swedish middle-aged 2,127 men and 3,100 women with baseline normal glucose tolerance, suggesting that psychological distress increases the risk of type 2 diabetes in Swedish men, but not in women (9). Kato et al. showed that the AOR for high stress compared with low stress was 1·36 (1·13–1·63) among men and 1·22 (0·98–1·51) among women (38). The effect of sex differences in PTSD on new-onset of diabetes mellitus remains largely unknown. One limited report for gender difference in the PTSD after the 911 attacks showed that male sex was not a risk factor for the association between PTSD and new-onset diabetes (AOR men 1·06 (0·96–1·17) vs. women 1·0 reference) (16).

To our knowledge, the current study is the first to show sex differences in the association between PTSD and new-onset diabetes. Our results also suggest that the post-disaster burden of probable depression and probable PTSD is causally related to new-onset diabetes in men but not in women.

### Potential mechanisms underlying the difference in probable depression- or PTSD-related new-onset diabetes

In the present study, the proportion of women among K6 ≥ 13 and PCL ≥ 44 groups was 70.6% and 67.1%, respectively; thus 2·40 and 2·04 times higher than that of men. This is consistent with previous studies showing that the incidence of PTSD is approximately twice as high in women as in men (39). Although the prevalence of probable depression and probable PTSD was higher in women, it was not a factor in developing diabetes mellitus in women but in men. There are four potential explanations for the sex difference in depression- or PTSD-related new-onset diabetes.

First, the symptom levels for probable depression and probable PTSD may differ between men and women. K6 and PCL-S are self-reported questionnaires and could be subjective. According to Eriksson et al., women were more likely to experience distress symptoms and overreport them, while men were more likely to tolerate distress symptoms and underreport them (9). If this is the case, men with distress symptoms may have larger neuroendocrine changes when the distress symptoms are self-reported (9). This notion agrees with our results showing that the frequencies of participants with probable depression or probable PTSD were lower in men, but a relationship between probable depression/PTSD and new-onset diabetes was present only in men.

Second, neuroendocrine networks, including the hypothalamic-pituitary-adrenal axis (HPA), oxidative stress, and sympathetic nerve activity during mental stress, can be modified, influenced, or both differentially in men and women (3, 39). The neuroendocrine network provides a structural and functional basis for interactions between the brain, hormones, and organs that allow individuals to respond to acute and chronic external stimuli (39). Trauma survivors with PTSD have a highly sensitized HPA axis characterized by decreased basal cortisol levels and increased negative feedback regulation of the HPA axis (40). The HPA axis is more sensitive and responds more strongly to acute stress in women than in men (41). Fonkoue et al. hypothesized that stress reactivity observed in men leads to a higher risk for new-onset diabetes *via* high levels of cortisol, while the lower cortisol response to stress observed in women stems from a hypo-reactivity of the HPA, which is associated with an increased risk for psychological distress and PTSD (39). Whether sex differences in the HPA are linked to sex differences in depression- and PTSD-related new-onset diabetes needs to be determined in future studies.

Third, the effects of probable depression and probable PTSD on physical activity and eating habits may differ between men and women. Physical inactivity and undesirable eating habits can result in obesity and a substantial risk of new-onset diabetes mellitus (42). Although correction of BMI could not abolish the impact of K6 ≥ 13 and PCL-S ≥ 44 on new-onset diabetes mellitus, sex differences in the distribution of abdominal and ectopic fat cannot be ruled out as a potential confounder for probable depression and probable PTSD-related new-onset diabetes.

Fourth, the association of psychological stress with employment rate, socioeconomic status, and education levels, which may differ between men and women, could be linked to gender difference in new-onset diabetes. In men, the age and sex-adjusted HR, but not multivariate-adjusted HR, was significant in change in work situation (**Tables 2A, B**). It has been reported that unemployment impairs mental health largely in men among evacuees of the Great East Japan Earthquake (43, 44). In contrast, the multivariate-adjusted HR of education ≥ 13 years was not significant in men but was significantly low in women. Collectively, change in work situation in men and education ≥ 13 years in women could be associated with gender difference in new-onset diabetes. Previous studies reported that higher education level was associated with lower diabetes risk (32, 45) in agreement with our finding in women. However, to our knowledge, there are no prior studies indicating gender difference in the association between education level and new-onset diabetes. We must wait for future studies and carefully interpret this phenomenon. The impact of socioeconomic status on diabetes onset can differ in men and women. However, we could not assess such relationship in this study because of a lack of individual socioeconomic sources. In men, the HR of K6 ≥ 13 and PCL-S ≥ 44 remained significant after correcting for psychosocial factors such as evacuation (Model 5), sleep satisfied (Model 6), education ≥ 13 years (Model 7), and change in work situation (Model 8). These results might support that probable depression and probable PTSD may be involved in onset of diabetes independently of the psychosocial factors measured in this study.

### Strength and limitation of this study

Our study has several strengths. The most notable are the longitudinal design and large sample size. Because the relationship between psychological burden and diabetes is bi-directional (4), establishing the order in which events occur is crucial, and providing insights into causal mechanisms and processes can be achieved only in a prospective and longitudinal manner. The next strength of this study was the use of annual investigations for new-onset diabetes mellitus by using the definition of objective indices, fasting plasma glucose level, HbA1c, or use of antihyperglycemic agents, not self-reported diabetes mellitus. By using these strengths of methodology, our study is the first to confirm the difference in men and women and the difference in the impacts of probable depression and probable PTSD on new-onset diabetes. Our study had several limitations. First, the current analyses did not account for potential confounding effects of antidepressant/anti-anxiety medications (46). Second, we could not determine probable depression before the Great East Japan Earthquake. Third, the lack of information on BMI, physical activity, and dietary records during the study period may be an important limitation. Although the baseline BMI, physical activity, and drinking status were not strong confounders, an increase in BMI caused by physical inactivity and hyperphagia (36, 37) through probable depression may be a confounder for the onset of diabetes. Fourth, we could not determine the underlying mechanism of sex differences in psychological burden-related new-onset diabetes. As discussed above, attenuation in the neuroendocrine network might be linked to sex differences. Fifth, we could not differentiate between the stressors for the onset of diabetes. These populations were survivors of the Great East Japan Earthquake, including the subsequent tsunami and the Fukushima Daiichi nuclear disaster; therefore, we could not differentiate the source of psychological burdens, such as post-traumatic stress response, chronic anxiety and guilt, ambiguous loss, family and community separation, and stigmatization. The radiation dose in the evacuation areas was substantially low, according to a report by the United Nations Scientific Committee on the Effects of Atomic Radiation (47). Therefore, the radiation-related direct effects on physical and mental health should be minimal, but the radiation-related psychological burden could be operative in the onset of diabetes. Sixth, it has been reported that objective measures are superior to subjective measures in assessing sleep as it relates to glycemic control (48, 49). We adopted satisfaction questionnaire for sleep assessment mainly for assessment of mental problems after the disaster and could not obtain objective measures such as sleep time primarily due to cost and questionnaire time. This may limit our interpretation on of the effects of sleep on onset of T2DM. Finally, we could not compare the incidence of type 2 diabetes between the participants in this study and the Japanese outside this area. Goto et al. estimated incidence rate of new-onset diabetes as 9.6 per 1000 person-years (95%CI 8.3-11.1) in pooled studies defining diabetes using laboratory data, not self-reported (50). The incidence rate of diabetes in the current study was all 19.6, men 27.5, and women 14.7 per 1000 person-years, suggesting that the incidence was largely higher in this cohort of participants. We need to find factors associated with this high-incident diabetes in future studies.

## Conclusion

**Figure 4:**
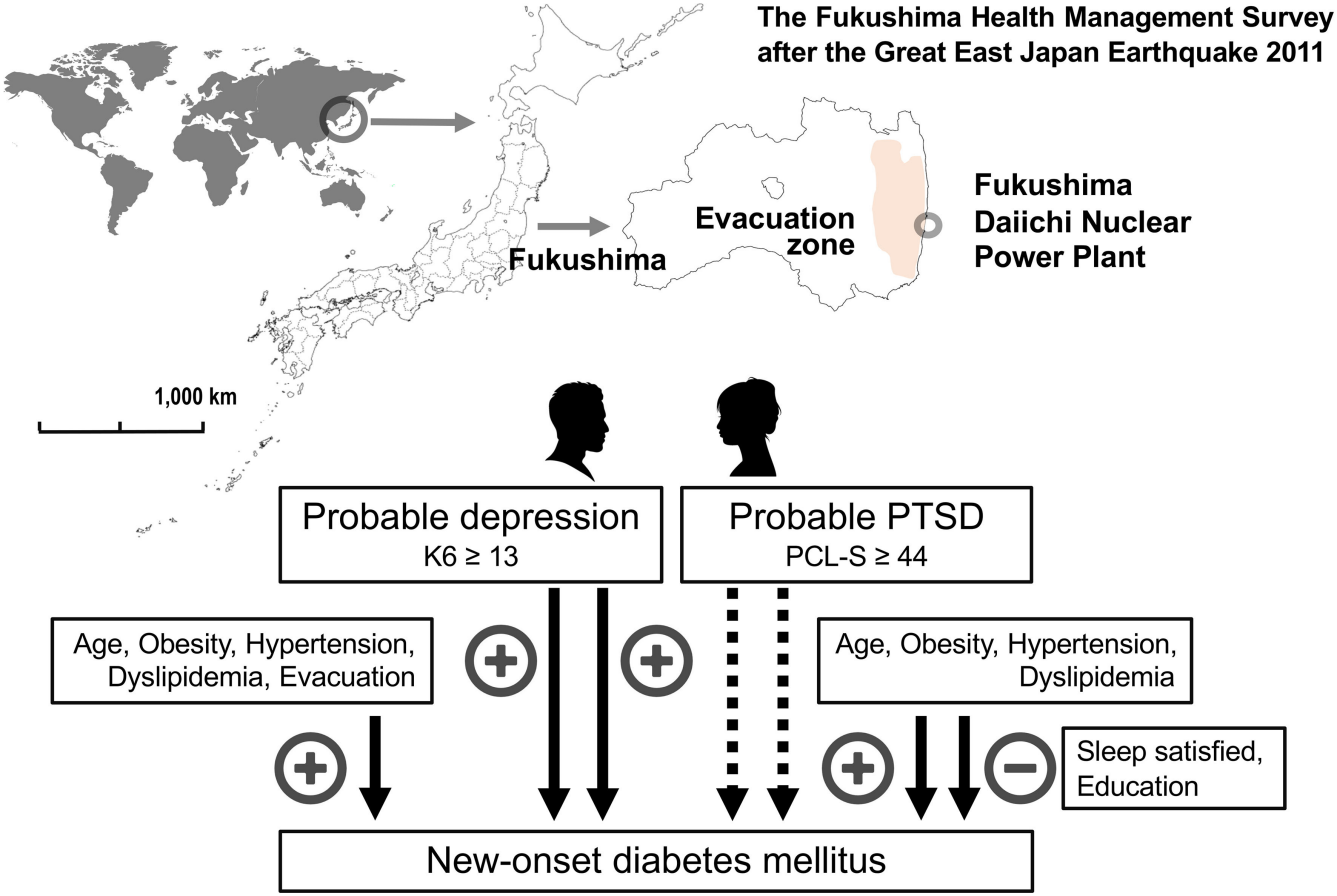
Graphic summary of the main findings of the article. The Fukushima Health Management Surve study targeted 123,314 people aged 40–74 years and was officially registered as being from 13 administrative districts at the time of the Great East Japan Earthquake 2011. Factors associated with new-onset diabetes mellitus in men and women were shown based on the Cox proportional hazards model after multivariate adjustment for established risk factors. Factors positively (+) and negatively (–) associated with new-onset diabetes mellitus were shown. Probable depression was defined as a Kessler 6 scale (K6) ≥ 13 and probable post-traumatic stress disorder (PTSD) as a PTSD Checklist—Stressor-Specific Version (PCL-S) ≥ 44.

In a 7-year longitudinal study conducted after the Great East Japan Earthquake, we found that psychological burden and PTSD were significant determinants for the onset of type 2 diabetes mellitus in the multivariate-adjusted model, but not after correction for evacuation, change in work situation, or sleep dissatisfaction. In men, but not women, psychological burden and PTSD were determined for new-onset diabetes independently of evacuation, change in work situation, or sleep dissatisfaction, indicating that the post-disaster psychological burden of probable depression and probable PTSD is causally related to new-onset diabetes in men, but not in women. Therefore, a prevention strategy for new-onset diabetes should consider sex differences in post-disaster circumstances. A graphic summary ot this article was shown in **Figure 4**.

## Data availability statement

The raw data supporting the conclusions of this article will be made available by the authors, without undue reservation.

## Ethics statement

The studies involving human participants were reviewed and approved by the Ethics Review Committee of Fukushima Medical University (#29064). The patients/participants provided their written informed consent to participate in this study.

## Author contributions

HH and MS contributed to the design of this study, conducted the analyses, and wrote the manuscript with inputs from all authors. MN performed the statistical analyses with input from TO, KO, HN, and FH. MN, TO, MM, KO, HH, FH, MHa, YS, AT, AS, JJK, MHo, HY, SY, HO, and KK were responsible for data collection and review of study procedures. All authors have read and approved the final version of the manuscript. MS is the guarantor of this work and, as such, has full access to all the data in the study and takes responsibility for the data integrity and accuracy of the data analysis. All authors contributed to the article and approved the submitted version.

## Funding

This survey was conducted as part of Fukushima Prefecture’s post-disaster recovery plans and was supported by the National Health Fund for Children and Adults Affected by the Nuclear Incident, Ministry of the Environment, Japan (MOEJ): fund number, n/a.

## Conflict of interest

The authors declare that the research was conducted in the absence of any commercial or financial relationships that could be construed as a potential conflict of interest.

## Publisher’s note

All claims expressed in this article are solely those of the authors and do not necessarily represent those of their affiliated organizations, or those of the publisher, the editors and the reviewers. Any product that may be evaluated in this article, or claim that may be made by its manufacturer, is not guaranteed or endorsed by the publisher.
